# Methyl 2-(2,2-dimethyl-3a,6a-di­hydro­furo[3,2-*d*][1,3]dioxol-5-yl)-4-oxo-4*H*-chromene-3-carboxyl­ate

**DOI:** 10.1107/S1600536813019648

**Published:** 2013-07-24

**Authors:** Zeenat Fatima, Thothadri Srinivasan, Jonnalagadda Naga Siva Rao, Raghavachary Raghunathan, Devadasan Velmurugan

**Affiliations:** a Centre of Advanced Study in Crystallography and Biophysics, University of Madras, Guindy Campus, Chennai 600 025, India; b Department of Organic Chemistry, University of Madras, Guindy Campus, Chennai 600 025, India

## Abstract

In the title mol­ecule, C_18_H_16_O_7_, the dioxolane ring adopts an envelope conformation with the dimethyl-substituted C atom as the flap. The furan ring is almost coplanar with the pyran ring, with a dihedral angle of 1.04 (10)° between the planes, and it makes a dihedral angle of 67.97 (11)° with the mean plane of the dioxolane ring. The latter makes a dihedral angle of 67.15 (10)° with the pyran ring. The O atom attached to the pyran ring deviates by −0.009 (1) Å. The crystal packing features C—H⋯O hydrogen bonds, forming a three-dimensional structure. The meth­oxy­carbonyl atoms are disordered over two positions, with a refined occupancy ratio of 0.508 (18):0.492 (18).

## Related literature

For the biological importance of 4*H*-chromene derivatives, see: Cai (2007 ▶, 2008 ▶); Cai *et al.* (2006 ▶); Caine (1993 ▶); Gabor (1988 ▶); Brooks (1998 ▶); Valenti *et al.* (1993 ▶); Hyana & Saimoto (1987 ▶); Tang *et al.* (2007 ▶). For conformational analysis, see: Cremer & Pople (1975 ▶).
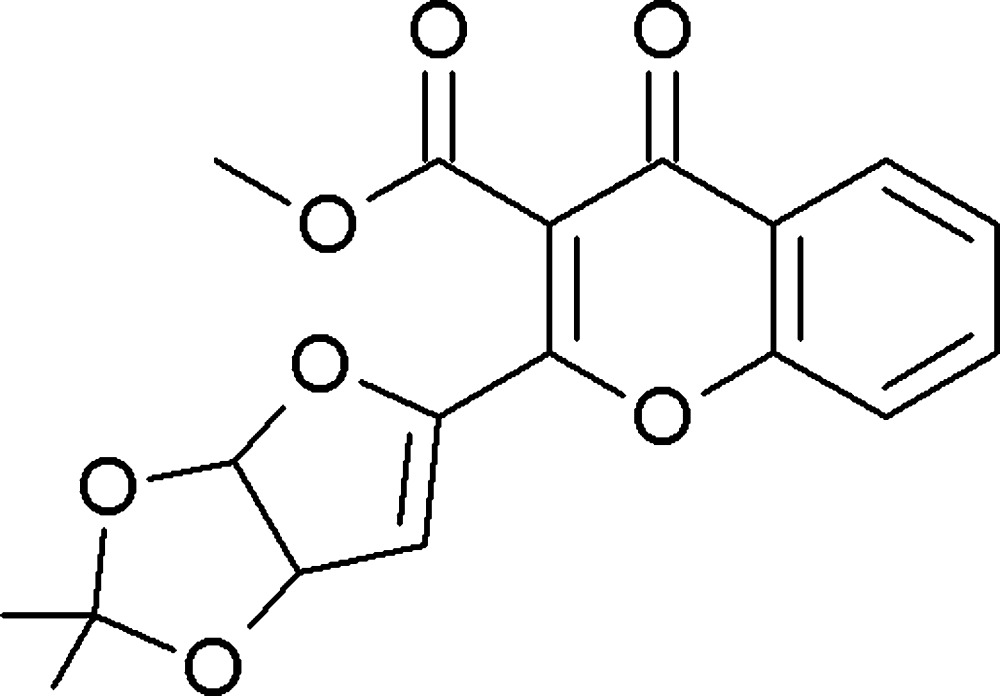


## Experimental

### 

#### Crystal data

C_18_H_16_O_7_
*M* *_r_* = 344.31
Orthorhombic, 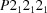

*a* = 6.8875 (3) Å
*b* = 15.4958 (6) Å
*c* = 15.9035 (6) Å
*V* = 1697.34 (12) Å^3^
*Z* = 4
Mo *K*α radiation
μ = 0.11 mm^−1^
*T* = 293 K
0.30 × 0.25 × 0.20 mm

#### Data collection

Bruker SMART APEXII area-detector diffractometer
Absorption correction: multi-scan (*SADABS*; Bruker, 2008 ▶) *T*_min_ = 0.969, *T*_max_ = 0.979
9565 measured reflections
4131 independent reflections
2983 reflections with *I* > 2σ(*I*)
*R*_int_ = 0.021

#### Refinement

*R*[*F* ^2^ > 2σ(*F* ^2^)] = 0.041
*wR*(*F* ^2^) = 0.108
*S* = 1.03
4131 reflections
267 parameters
99 restraints
H-atom parameters constrained
Δρ_max_ = 0.18 e Å^−3^
Δρ_min_ = −0.19 e Å^−3^

### 

Data collection: *APEX2* (Bruker, 2008 ▶); cell refinement: *SAINT* (Bruker, 2008 ▶); data reduction: *SAINT*; program(s) used to solve structure: *SHELXS97* (Sheldrick, 2008 ▶); program(s) used to refine structure: *SHELXL97* (Sheldrick, 2008 ▶); molecular graphics: *ORTEP-3 for Windows* (Farrugia, 2012 ▶); software used to prepare material for publication: *SHELXL97* (Sheldrick, 2008 ▶) and *PLATON* (Spek, 2009 ▶).

## Supplementary Material

Crystal structure: contains datablock(s) global, I. DOI: 10.1107/S1600536813019648/su2622sup1.cif

Structure factors: contains datablock(s) I. DOI: 10.1107/S1600536813019648/su2622Isup2.hkl

Supplementary material file. DOI: 10.1107/S1600536813019648/su2622Isup3.cml

Additional supplementary materials:  crystallographic information; 3D view; checkCIF report

## Figures and Tables

**Table 1:** Hydrogen-bond geometry (Å, °)

*D*—H⋯*A*	*D*—H	H⋯*A*	*D*⋯*A*	*D*—H⋯*A*
C6—H6⋯O7^i^	0.93	2.59	3.296 (2)	133
C13—H13⋯O3^ii^	0.93	2.59	3.230 (10)	127
C14—H14⋯O1^ii^	0.98	2.50	3.429 (2)	159
C18—H18*C*⋯O1^iii^	0.96	2.55	3.460 (3)	159

Symmetry codes: (i) 
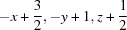
; (ii) 
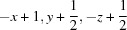
; (iii) 
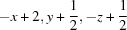
.

**Fig. 1.**
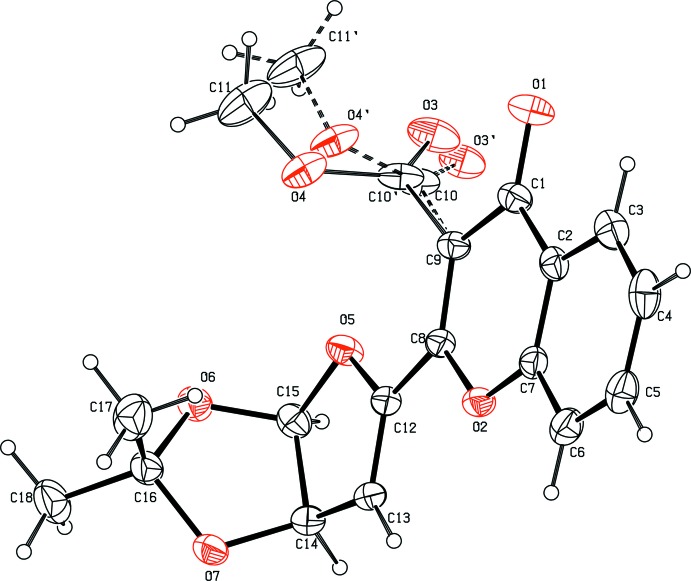
The molecular structure of the title molecule, with the atom labelling. Displacement ellipsoids are drawn at the 30% probability level.

**Fig. 2.**
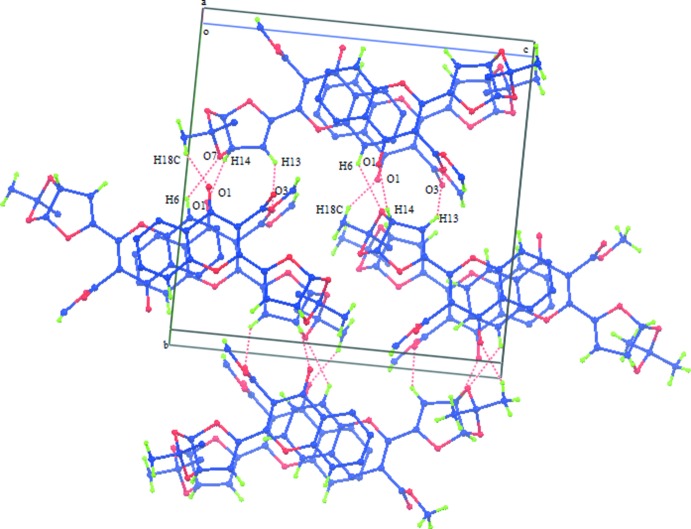
The crystal packing of the title compound viewed along the a axis. C-H···O hydrogen bonds are shown as dashed lines (see Table 1 for details).

## supplementary crystallographic information

### Comment

4*H*-Chromenes are biologically important compounds used as synthetic ligands in the design of drugs and discovery processes. They exhibit numerous biological and pharmacological properties, such as anti-viral, anti-fungal, antiinflammatory, anti-diabetic, cardionthonic, anti-anaphylactic and anti-cancer activity (Cai, 2008, 2007; Cai *et al.*, 2006; Gabor, 1988; Brooks, 1998; Valenti *et al.*, 1993; Hyana & Saimoto, 1987; Tang *et al.*, 2007). In view of the different applications of this class of compounds, we have undertaken the synthesis and the crystal structure analysis of the title compound.

In the title molecule, Fig. 1, the dioxolane ring adopts an *envelope* conformation with atom C16 as the flap. The furan ring (O5/C12—C15) is almost coplanar with the pyran ring (O2/C1/C2/C7—C9), with a dihedral angle of 1.04 (10) ° and makes a dihedral angle of 67.97 (11) ° with the mean plane of the dioxolane ring (06/07/C14—C16). The mean plane of the dioxolane ring makes a dihedral angle of 67.15 (10) ° with the pyran ring. The oxygen atom O1 attached with the pyran ring deviates by -0.009 (1) Å. The methyl carbon atoms C17 and C18 attached with the dioxolane ring deviate by -1.666 (3) Å and 0.736 (3) Å, respectively from the mean plane.

The crystal packing is stabilized by intermolecular C—H···O hydrogen bonds, forming a three-dimensional structure (Table 1 and Fig. 2).

### Experimental

Triethylamine (1.10 ml, 4 equiv) was added to a stirred solution of 4-hydroxycoumarin (0.32 g, 2 mmol) and (*E*)-6-(benzyloxy)-2,2-dimethyl-5- (2-nitrovinyl)tetrahydrofuro[3,2-*d*][1,3]dioxole (0.65 g, 4 mmol) in methanol (6 ml). The reaction mixture was heated at 343 - 353 K for 24 h, and the progress of the reaction was monitored by TLC. After completion of the reaction, the solvent was evaporated in vacuum. The resulting residue was further purifed by flash column chromatography (ethyl acetate/hexane) on silica gel. Single crystals suitable for X-ray diffraction were obtained by slow evaporation of a solution of the title compound in ethyl acetate at room temperature.

### Refinement

The methyl carboxylate group (O3/O4/C10/C11) is disordered over two positions with arefined occupancy ratio of 0.508 (18):0.492 (18). The C9—C10 and C9'— C10' interatomic distances were restrained to be equal using the *SHELXL97* SADI command. The *SHELXL97 DFIX* instruction restrains the interatomic distance between pairs of atoms C10—O4/C10'—O4', O4—C11/O4'—C11' and C10—O3/C10'—O3' to 1.40 (1), 1.45 (1) and 1.20 (1) Å, respectively. The restraints, *SHELXL97* commands FLAT and SIMU, ensure chemically and physically reasonable parameters for the disordered atoms. The hydrogen atoms were placed in calculated positions and treated as riding atoms: C—H = 0.93 - 0.98 Å with *U*_iso_(H) = 1.5U_eq_(C) for methyl H atoms and = 1.2U_eq_(C) for other H atoms.

### Crystal data

**Table:**

C_18_H_16_O_7_	*F*(000) = 720
*M**_r_* = 344.31	*D*_x_ = 1.347 Mg m^−^^3^
Orthorhombic, *P*2_1_2_1_2_1_	Mo *K*α radiation, λ = 0.71073 Å
Hall symbol: P 2ac 2ab	Cell parameters from 4131 reflections
*a* = 6.8875 (3) Å	θ = 1.8–28.3°
*b* = 15.4958 (6) Å	µ = 0.11 mm^−^^1^
*c* = 15.9035 (6) Å	*T* = 293 K
*V* = 1697.34 (12) Å^3^	Block, colourless
*Z* = 4	0.30 × 0.25 × 0.20 mm

### Data collection

**Table:**

Bruker SMART APEXII area-detector diffractometer	4131 independent reflections
Radiation source: fine-focus sealed tube	2983 reflections with *I* > 2σ(*I*)
Graphite monochromator	*R*_int_ = 0.021
ω and φ scans	θ_max_ = 28.3°, θ_min_ = 1.8°
Absorption correction: multi-scan (*SADABS*; Bruker, 2008)	*h* = −9→9
*T*_min_ = 0.969, *T*_max_ = 0.979	*k* = −20→14
9565 measured reflections	*l* = −14→21

### Refinement

**Table:**

Refinement on *F*^2^	Primary atom site location: structure-invariant direct methods
Least-squares matrix: full	Secondary atom site location: difference Fourier map
*R*[*F*^2^ > 2σ(*F*^2^)] = 0.041	Hydrogen site location: inferred from neighbouring sites
*wR*(*F*^2^) = 0.108	H-atom parameters constrained
*S* = 1.03	*w* = 1/[σ^2^(*F*_o_^2^) + (0.0575*P*)^2^ + 0.0367*P*] where *P* = (*F*_o_^2^ + 2*F*_c_^2^)/3
4131 reflections	(Δ/σ)_max_ < 0.001
267 parameters	Δρ_max_ = 0.18 e Å^−^^3^
99 restraints	Δρ_min_ = −0.19 e Å^−^^3^

### Special details

**Table:**

Geometry. All e.s.d.'s (except the e.s.d. in the dihedral angle between two l.s. planes) are estimated using the full covariance matrix. The cell e.s.d.'s are taken into account individually in the estimation of e.s.d.'s in distances, angles and torsion angles; correlations between e.s.d.'s in cell parameters are only used when they are defined by crystal symmetry. An approximate (isotropic) treatment of cell e.s.d.'s is used for estimating e.s.d.'s involving l.s. planes.
Refinement. Refinement of *F*^2^ against ALL reflections. The weighted *R*-factor *wR* and goodness of fit *S* are based on *F*^2^, conventional *R*-factors *R* are based on *F*, with *F* set to zero for negative *F*^2^. The threshold expression of *F*^2^ > σ(*F*^2^) is used only for calculating *R*-factors(gt) *etc*. and is not relevant to the choice of reflections for refinement. *R*-factors based on *F*^2^ are statistically about twice as large as those based on *F*, and *R*- factors based on ALL data will be even larger.

### Fractional atomic coordinates and isotropic or equivalent isotropic displacement parameters (Å^2^)

**Table:**

	*x*	*y*	*z*	*U*_iso_*/*U*_eq_	Occ. (<1)
C1	0.6753 (3)	0.14269 (11)	0.41547 (11)	0.0497 (4)	
C2	0.6727 (3)	0.20723 (11)	0.48234 (11)	0.0476 (4)	
C3	0.6773 (3)	0.18438 (14)	0.56749 (12)	0.0627 (5)	
H3	0.6789	0.1265	0.5829	0.075*	
C4	0.6797 (3)	0.24736 (17)	0.62848 (13)	0.0720 (6)	
H4	0.6828	0.2316	0.6849	0.086*	
C5	0.6775 (3)	0.33337 (15)	0.60685 (13)	0.0666 (5)	
H5	0.6799	0.3754	0.6486	0.080*	
C6	0.6719 (3)	0.35725 (13)	0.52421 (12)	0.0595 (5)	
H6	0.6703	0.4153	0.5095	0.071*	
C7	0.6686 (3)	0.29417 (11)	0.46269 (10)	0.0456 (4)	
C8	0.6653 (2)	0.26426 (9)	0.31685 (10)	0.0425 (4)	
C9	0.6757 (3)	0.17837 (10)	0.32992 (11)	0.0449 (4)	
C12	0.6554 (3)	0.30873 (10)	0.23675 (10)	0.0449 (4)	
C13	0.6472 (3)	0.39241 (11)	0.22124 (11)	0.0520 (4)	
H13	0.6440	0.4357	0.2617	0.062*	
C14	0.6441 (3)	0.40610 (11)	0.12871 (12)	0.0563 (5)	
H14	0.5286	0.4381	0.1108	0.068*	
C15	0.6451 (3)	0.31385 (11)	0.09405 (11)	0.0545 (4)	
H15	0.5272	0.3025	0.0614	0.065*	
C16	0.9405 (3)	0.37635 (12)	0.06399 (13)	0.0614 (5)	
C17	1.0874 (4)	0.34751 (18)	0.12818 (18)	0.0877 (8)	
H17A	1.1695	0.3952	0.1430	0.132*	
H17B	1.1650	0.3018	0.1051	0.132*	
H17C	1.0214	0.3271	0.1775	0.132*	
C18	1.0317 (5)	0.40685 (17)	−0.01674 (15)	0.0937 (9)	
H18A	0.9318	0.4186	−0.0573	0.141*	
H18B	1.1166	0.3629	−0.0381	0.141*	
H18C	1.1048	0.4585	−0.0063	0.141*	
O1	0.6772 (2)	0.06461 (8)	0.42838 (9)	0.0699 (4)	
O2	0.66072 (19)	0.32230 (7)	0.38098 (7)	0.0491 (3)	
O5	0.6553 (2)	0.25723 (7)	0.16682 (7)	0.0544 (3)	
O6	0.8081 (2)	0.30677 (8)	0.04404 (8)	0.0618 (4)	
O7	0.8187 (2)	0.44315 (7)	0.09562 (8)	0.0653 (4)	
O3	0.5952 (18)	0.0645 (6)	0.2327 (6)	0.081 (2)	0.508 (18)
O4	0.8963 (12)	0.1191 (6)	0.2314 (5)	0.0710 (16)	0.508 (18)
C10	0.7106 (15)	0.1130 (7)	0.2611 (7)	0.0565 (18)	0.508 (18)
C11	0.9518 (18)	0.0578 (9)	0.1669 (7)	0.108 (3)	0.508 (18)
H11A	0.9967	0.0056	0.1929	0.163*	0.508 (18)
H11B	1.0539	0.0820	0.1332	0.163*	0.508 (18)
H11C	0.8417	0.0453	0.1320	0.163*	0.508 (18)
O3'	0.5216 (14)	0.0826 (5)	0.2331 (6)	0.0666 (17)	0.492 (18)
O4'	0.8513 (16)	0.0905 (6)	0.2447 (5)	0.0742 (17)	0.492 (18)
C10'	0.6656 (16)	0.1144 (8)	0.2596 (7)	0.061 (2)	0.492 (18)
C11'	0.867 (2)	0.0196 (8)	0.1862 (6)	0.105 (4)	0.492 (18)
H11D	0.8325	−0.0332	0.2140	0.158*	0.492 (18)
H11E	0.9983	0.0157	0.1661	0.158*	0.492 (18)
H11F	0.7812	0.0291	0.1396	0.158*	0.492 (18)

### Atomic displacement parameters (Å^2^)

**Table:**

	*U*^11^	*U*^22^	*U*^33^	*U*^12^	*U*^13^	*U*^23^
C1	0.0482 (9)	0.0419 (9)	0.0589 (10)	−0.0049 (8)	−0.0052 (9)	0.0058 (8)
C2	0.0378 (8)	0.0541 (10)	0.0508 (9)	−0.0047 (8)	0.0026 (8)	0.0037 (8)
C3	0.0542 (11)	0.0733 (13)	0.0606 (12)	−0.0067 (11)	−0.0008 (10)	0.0108 (10)
C4	0.0567 (12)	0.1145 (19)	0.0448 (10)	−0.0050 (14)	0.0063 (10)	0.0005 (11)
C5	0.0576 (11)	0.0838 (15)	0.0584 (11)	−0.0008 (11)	0.0093 (11)	−0.0180 (10)
C6	0.0542 (10)	0.0625 (11)	0.0618 (11)	0.0016 (10)	0.0070 (10)	−0.0129 (9)
C7	0.0377 (8)	0.0492 (9)	0.0500 (9)	−0.0010 (8)	0.0055 (8)	−0.0024 (7)
C8	0.0407 (8)	0.0360 (8)	0.0507 (9)	−0.0020 (7)	0.0028 (8)	−0.0020 (7)
C9	0.0480 (8)	0.0358 (8)	0.0508 (9)	−0.0021 (8)	−0.0031 (9)	−0.0011 (7)
C12	0.0442 (9)	0.0389 (8)	0.0515 (9)	−0.0010 (7)	0.0049 (8)	−0.0015 (7)
C13	0.0616 (11)	0.0377 (8)	0.0568 (10)	0.0088 (8)	0.0132 (9)	0.0027 (7)
C14	0.0591 (11)	0.0453 (9)	0.0643 (11)	0.0097 (9)	0.0072 (9)	0.0115 (8)
C15	0.0566 (11)	0.0554 (10)	0.0515 (10)	−0.0042 (9)	−0.0036 (9)	0.0078 (8)
C16	0.0721 (13)	0.0455 (10)	0.0665 (12)	−0.0116 (9)	0.0215 (11)	−0.0097 (9)
C17	0.0602 (13)	0.0983 (18)	0.1045 (19)	−0.0049 (13)	0.0050 (14)	−0.0084 (16)
C18	0.130 (2)	0.0729 (14)	0.0783 (16)	−0.0319 (15)	0.0492 (15)	−0.0167 (13)
O1	0.0924 (10)	0.0408 (7)	0.0765 (9)	−0.0053 (7)	−0.0154 (9)	0.0126 (6)
O2	0.0601 (7)	0.0378 (5)	0.0495 (6)	0.0006 (6)	0.0059 (6)	−0.0029 (5)
O5	0.0746 (9)	0.0394 (6)	0.0491 (7)	−0.0077 (6)	−0.0002 (6)	0.0004 (5)
O6	0.0768 (9)	0.0494 (7)	0.0592 (7)	−0.0103 (7)	0.0143 (7)	−0.0078 (6)
O7	0.0865 (10)	0.0385 (6)	0.0709 (8)	−0.0073 (7)	0.0242 (8)	−0.0005 (6)
O3	0.110 (5)	0.047 (3)	0.087 (3)	−0.023 (3)	−0.030 (4)	−0.002 (2)
O4	0.081 (3)	0.064 (3)	0.068 (3)	0.025 (3)	−0.010 (2)	−0.017 (3)
C10	0.081 (4)	0.031 (3)	0.057 (3)	0.002 (3)	−0.029 (3)	−0.002 (3)
C11	0.126 (7)	0.109 (7)	0.090 (5)	0.048 (5)	−0.009 (4)	−0.041 (5)
O3'	0.087 (4)	0.047 (3)	0.066 (2)	−0.015 (3)	−0.022 (3)	−0.003 (2)
O4'	0.097 (4)	0.056 (3)	0.069 (3)	0.031 (3)	−0.013 (3)	−0.020 (2)
C10'	0.087 (4)	0.037 (3)	0.058 (3)	0.008 (3)	−0.013 (3)	0.005 (3)
C11'	0.151 (9)	0.094 (6)	0.072 (4)	0.060 (5)	−0.021 (5)	−0.039 (4)

### Geometric parameters (Å, º)

**Table:**

C1—O1	1.227 (2)	C15—O6	1.381 (2)
C1—C2	1.460 (2)	C15—O5	1.454 (2)
C1—C9	1.469 (2)	C15—H15	0.9800
C2—C7	1.383 (2)	C16—O7	1.424 (2)
C2—C3	1.400 (3)	C16—O6	1.447 (2)
C3—C4	1.376 (3)	C16—C17	1.505 (3)
C3—H3	0.9300	C16—C18	1.505 (3)
C4—C5	1.377 (3)	C17—H17A	0.9600
C4—H4	0.9300	C17—H17B	0.9600
C5—C6	1.366 (3)	C17—H17C	0.9600
C5—H5	0.9300	C18—H18A	0.9600
C6—C7	1.383 (2)	C18—H18B	0.9600
C6—H6	0.9300	C18—H18C	0.9600
C7—O2	1.372 (2)	O3—C10	1.183 (7)
C8—C9	1.349 (2)	O4—C10	1.366 (7)
C8—O2	1.3602 (19)	O4—C11	1.449 (7)
C8—C12	1.450 (2)	C11—H11A	0.9600
C9—C10'	1.496 (6)	C11—H11B	0.9600
C9—C10	1.511 (5)	C11—H11C	0.9600
C12—C13	1.321 (2)	O3'—C10'	1.185 (7)
C12—O5	1.3689 (19)	O4'—C10'	1.353 (7)
C13—C14	1.487 (3)	O4'—C11'	1.444 (7)
C13—H13	0.9300	C11'—H11D	0.9600
C14—O7	1.433 (2)	C11'—H11E	0.9600
C14—C15	1.532 (3)	C11'—H11F	0.9600
C14—H14	0.9800		
			
O1—C1—C2	123.62 (16)	O6—C15—O5	111.78 (15)
O1—C1—C9	121.75 (16)	O6—C15—C14	106.56 (14)
C2—C1—C9	114.64 (14)	O5—C15—C14	106.08 (14)
C7—C2—C3	117.73 (17)	O6—C15—H15	110.8
C7—C2—C1	120.20 (16)	O5—C15—H15	110.8
C3—C2—C1	122.07 (17)	C14—C15—H15	110.8
C4—C3—C2	120.18 (19)	O7—C16—O6	104.35 (16)
C4—C3—H3	119.9	O7—C16—C17	111.85 (17)
C2—C3—H3	119.9	O6—C16—C17	110.54 (17)
C3—C4—C5	120.69 (19)	O7—C16—C18	108.59 (18)
C3—C4—H4	119.7	O6—C16—C18	108.04 (17)
C5—C4—H4	119.7	C17—C16—C18	113.0 (2)
C6—C5—C4	120.20 (19)	C16—C17—H17A	109.5
C6—C5—H5	119.9	C16—C17—H17B	109.5
C4—C5—H5	119.9	H17A—C17—H17B	109.5
C5—C6—C7	119.3 (2)	C16—C17—H17C	109.5
C5—C6—H6	120.3	H17A—C17—H17C	109.5
C7—C6—H6	120.3	H17B—C17—H17C	109.5
O2—C7—C6	116.50 (16)	C16—C18—H18A	109.5
O2—C7—C2	121.61 (15)	C16—C18—H18B	109.5
C6—C7—C2	121.88 (17)	H18A—C18—H18B	109.5
C9—C8—O2	122.55 (15)	C16—C18—H18C	109.5
C9—C8—C12	127.36 (14)	H18A—C18—H18C	109.5
O2—C8—C12	110.09 (12)	H18B—C18—H18C	109.5
C8—C9—C1	120.94 (15)	C8—O2—C7	119.96 (12)
C8—C9—C10'	122.4 (6)	C12—O5—C15	107.15 (12)
C1—C9—C10'	116.3 (6)	C15—O6—C16	109.09 (13)
C8—C9—C10	123.9 (6)	C16—O7—C14	109.45 (12)
C1—C9—C10	114.8 (6)	C10—O4—C11	116.5 (6)
C10'—C9—C10	11.9 (6)	O3—C10—O4	122.8 (7)
C13—C12—O5	114.86 (15)	O3—C10—C9	126.5 (8)
C13—C12—C8	129.24 (15)	O4—C10—C9	110.6 (6)
O5—C12—C8	115.90 (12)	C10'—O4'—C11'	113.1 (7)
C12—C13—C14	108.99 (15)	O3'—C10'—O4'	128.0 (7)
C12—C13—H13	125.5	O3'—C10'—C9	125.5 (8)
C14—C13—H13	125.5	O4'—C10'—C9	105.6 (6)
O7—C14—C13	114.10 (17)	O4'—C11'—H11D	109.5
O7—C14—C15	103.77 (14)	O4'—C11'—H11E	109.5
C13—C14—C15	102.88 (14)	H11D—C11'—H11E	109.5
O7—C14—H14	111.8	O4'—C11'—H11F	109.5
C13—C14—H14	111.8	H11D—C11'—H11F	109.5
C15—C14—H14	111.8	H11E—C11'—H11F	109.5
			
O1—C1—C2—C7	−179.32 (18)	O7—C14—C15—O5	117.25 (16)
C9—C1—C2—C7	0.8 (2)	C13—C14—C15—O5	−1.91 (19)
O1—C1—C2—C3	1.7 (3)	C9—C8—O2—C7	0.6 (2)
C9—C1—C2—C3	−178.20 (17)	C12—C8—O2—C7	−179.96 (14)
C7—C2—C3—C4	−0.6 (3)	C6—C7—O2—C8	177.25 (15)
C1—C2—C3—C4	178.34 (17)	C2—C7—O2—C8	−2.9 (2)
C2—C3—C4—C5	0.0 (3)	C13—C12—O5—C15	−0.2 (2)
C3—C4—C5—C6	0.4 (4)	C8—C12—O5—C15	−179.94 (15)
C4—C5—C6—C7	−0.1 (3)	O6—C15—O5—C12	117.13 (16)
C5—C6—C7—O2	179.26 (18)	C14—C15—O5—C12	1.38 (19)
C5—C6—C7—C2	−0.6 (3)	O5—C15—O6—C16	−98.06 (17)
C3—C2—C7—O2	−178.92 (16)	C14—C15—O6—C16	17.39 (19)
C1—C2—C7—O2	2.1 (3)	O7—C16—O6—C15	−26.20 (19)
C3—C2—C7—C6	1.0 (3)	C17—C16—O6—C15	94.21 (18)
C1—C2—C7—C6	−178.04 (17)	C18—C16—O6—C15	−141.63 (19)
O2—C8—C9—C1	2.4 (3)	O6—C16—O7—C14	24.8 (2)
C12—C8—C9—C1	−176.98 (17)	C17—C16—O7—C14	−94.74 (19)
O2—C8—C9—C10'	175.3 (5)	C18—C16—O7—C14	139.83 (19)
C12—C8—C9—C10'	−4.1 (6)	C13—C14—O7—C16	96.76 (18)
O2—C8—C9—C10	−170.6 (5)	C15—C14—O7—C16	−14.40 (19)
C12—C8—C9—C10	10.0 (5)	C11—O4—C10—O3	−4.9 (16)
O1—C1—C9—C8	177.14 (18)	C11—O4—C10—C9	177.9 (8)
C2—C1—C9—C8	−2.9 (3)	C8—C9—C10—O3	−107.4 (13)
O1—C1—C9—C10'	3.8 (5)	C1—C9—C10—O3	79.2 (13)
C2—C1—C9—C10'	−176.3 (5)	C10'—C9—C10—O3	−21 (4)
O1—C1—C9—C10	−9.3 (5)	C8—C9—C10—O4	69.7 (11)
C2—C1—C9—C10	170.7 (4)	C1—C9—C10—O4	−103.7 (10)
C9—C8—C12—C13	−179.15 (19)	C10'—C9—C10—O4	156 (6)
O2—C8—C12—C13	1.4 (3)	C11'—O4'—C10'—O3'	2.9 (18)
C9—C8—C12—O5	0.5 (3)	C11'—O4'—C10'—C9	171.8 (8)
O2—C8—C12—O5	−178.90 (14)	C8—C9—C10'—O3'	−90.0 (14)
O5—C12—C13—C14	−1.1 (2)	C1—C9—C10'—O3'	83.2 (14)
C8—C12—C13—C14	178.57 (17)	C10—C9—C10'—O3'	169 (7)
C12—C13—C14—O7	−109.86 (17)	C8—C9—C10'—O4'	100.7 (10)
C12—C13—C14—C15	1.8 (2)	C1—C9—C10'—O4'	−86.1 (10)
O7—C14—C15—O6	−1.99 (18)	C10—C9—C10'—O4'	−1 (5)
C13—C14—C15—O6	−121.15 (16)		

### Hydrogen-bond geometry (Å, º)

**Table:**

*D*—H···*A*	*D*—H	H···*A*	*D*···*A*	*D*—H···*A*
C6—H6···O7^i^	0.93	2.59	3.296 (2)	133
C13—H13···O3^ii^	0.93	2.59	3.230 (10)	127
C14—H14···O1^ii^	0.98	2.50	3.429 (2)	159
C18—H18*C*···O1^iii^	0.96	2.55	3.460 (3)	159

Symmetry codes: (i) −*x*+3/2, −*y*+1, *z*+1/2; (ii) −*x*+1, *y*+1/2, −*z*+1/2; (iii) −*x*+2, *y*+1/2, −*z*+1/2.
